# Factors Associated With the Development of Chronic Kidney Disease in Children With Congenital Anomalies of the Kidney and Urinary Tract

**DOI:** 10.3389/fped.2020.00298

**Published:** 2020-06-15

**Authors:** Saskia Isert, Dominik Müller, Julia Thumfart

**Affiliations:** ^1^Department of Pediatric Gastroenterology, Nephrology and Metabolic Diseases, Charité - Universitätsmedizin Berlin, Berlin, Germany; ^2^Department of Pediatrics, Helios Klinikum Berlin-Buch, Berlin, Germany

**Keywords:** CAKUT, chronic kidney disease, children, genetic, prenatal factors

## Abstract

Congenital anomalies of the kidney and urinary tract (CAKUT) are the most common cause of end-stage renal disease in children and adolescents. The diversity of the malformations summarized by CAKUT is high and there are numerous associated syndromes. The genetic background of these malformations remains unknown in the majority of cases. The aim of this study was to evaluate factors associated with the development of chronic kidney disease (CKD) and underlying genetic aberrations in children and adolescents with CAKUT. For this purpose, data from patients with CAKUT presented at the pediatric nephrology outpatient clinic were analyzed in a cross-sectional single-center study. Among the 405 patients, the commonest findings related to CAKUT were renal hypoplasia/dysplasia (65%), followed by hydronephrosis (43%). Forty-four percent of the patients were suffering from CKD, 6% were ranked as end-stage renal disease. In the univariate analysis, male gender and premature birth were associated with higher CKD stages (*p* = 0.004 resp. *p* < 0.001). Children with an abnormal prenatal ultrasound had more often a glomerular filtration rate of <30 ml/min/1.73 m^2^ (*p* = 0.004). Patients with urinary tract infections as first symptom whereas had significant lower CKD stages (*p* = 0,006). In the multivariate analysis, premature birth (*p* = 0.033) and urinary tract infection as the first symptom (*p* = 0.043) were significantly associated with CKD stage ≥ II. Among the 16% of the children who have undergone genetic analyses the most frequent genetic aberration was a mutation in *HNF1*β-gene. These results can be used for the care of patients with CAKUT subject to factors associated with developing CKD.

## Introduction

Congenital anomalies of the kidney and urinary tract (CAKUT) comprise a range of abnormalities resulting from defects in morphogenesis of the kidney and/or urinary tract (1). However, the term CAKUT is not well defined including for example renal dysplasias, hypoplasias, agenesis, horseshoe kidneys, obstructions at the ureteropelvic junction or at the vesicoureteral junction, posterior urethral valves (PUV), and vesicoureteral reflux (VUR) (2, 3). Many of the entities that are categorized as CAKUT are rare kidney diseases.

CAKUT manifest isolated or as part of a syndrome contributing to heterogeneity of the affected population (2). Phenotypically, CAKUT range from asymptomatic cases to chronic kidney disease (CKD) (4). CAKUT as disease group represent the most common reason for end-stage renal disease (ESRD) in children and adolescents (5–8).

The diagnosis CAKUT can already be made within the fetal ultrasound scans (4). Impaired urine production due to severe kidney dysplasia or obstruction in the urinary tract can cause an oligo- or anhydramnios (9). Initial symptoms of CAKUT are urinary tract infections (UTI), arterial hypertension or an abdominal swelling due to an enlarged kidney or urinary bladder (3, 4). As a considerable part of the patients stays asymptomatic they become apparent when imaging methods are applied because of a different issue (10).

The genetic diagnostic is very complex due to numerous involved genes and the variable genotype-phenotype-correlation (10, 11). More than 20 genes are currently known in which mutations can cause monogenic CAKUT (12). Nevertheless, in the majority of cases genetic defects responsible for the abnormalities cannot be identified (10). There is still a lack of knowledge about how to assess different patient's aspects, because the phenotypes and genotypes of patients with CAKUT are so diverse.

The prognosis of the patients varies considerably as in some patients the ultrasound findings return to normal after some time (13). On the other hand, CAKUT are with about 40% the leading cause for ESRD in childhood and adolescence (5–8). Up to now, little is known about which factors influence the prognosis of children and adolescents with CAKUT.

The aim of this study was to identify factors associated with the development of CKD and underlying genetic aberrations in children and adolescents with CAKUT.

## Materials and Methods

Patient charts of children and adolescents with CAKUT presented between January 1st, 2014, and September 1st, 2015, at the pediatric nephrology outpatient clinic of the Charité - Universitätsmedizin Berlin (Germany) were retrospectively analyzed as a cross-sectional study. Patients with an isolated double kidney were excluded. The following items were analyzed: gender, age, diagnoses, first symptoms, ultrasound findings, stage of CKD, secondary findings/diagnoses and genetic testing. In 271 patients, no information on at least one item could be found in the patient file.

The age of each patient was calculated on the reference date September 1st, 2015. For classifying of the renal function the current GFR was used. The GFR was assessed in most cases by calculation with the revised Schwartz formula (14) or, if available, by cystatin C concentration. Patients were ranked in CKD stage I if they had bilateral pathologic ultrasound findings or in case of unilateral findings if the healthy kidney had a size below the 50th percentile. Current renal function was also used to determine any relationship between CKD and possible associated factors.

Patients with prenatal abnormalities of the kidney or urinary tract or an oligo-/anhydramnios were classified as prenatally abnormal. Of the ultrasound results, the latest ones were chosen for analysis except for patients after kidney transplantation. In these cases, reports prior to transplantation were used.

Patients for genetic testing were selected based on physician's decision mainly influenced by severity of CKD, family history and presence of syndromic disease. Documented genetic investigation methods were chromosome analysis, array-based comparative genomic hybridization, fluorescence *in situ* hybridization, Sanger sequencing and next generation sequencing. The latest next generation sequencing panels included the following genes associated with CAKUT: *ACE, AGT, AGTR1, BICC1, BMP4, CDC5L, CHD1L, DSTYK, EYA1, FANCB, FREM1, FREM2, FRAS1, GATA3, GDNF, GLIS3, HNF1*β, *ITGA8, LHX1, PAX2, PAX8, REN, RET, ROBO2, SALL1, SALL4, SIX1, SIX2, SIX5, SLIT2, SOX17, TBX18, UPK2, UPK3A, WNT4*, and *WWTR1*. Genetic analysis and interpretation were done by human genetic laboratories (e.g. Bioscientia Institute for Medical Diagnostics GmbH and Institute of Medical Genetics and Human Genetics of the Charité - Universitätsmedizin Berlin**)**.

### Statistical Analysis

Descriptive analysis was done by use of Microsoft Excel 2013. Significance tests were done by use of IBM SPSS Statistics 24. The chi-square test was used for nominal scaled data and the Mann-Whitney-U-Test for ordinal scaled or not normally distributed metrical scaled data. Normal distribution was tested with the Shapiro-Wilk-Test. Due to non-normal distribution of the patient's age the median was calculated with respect to its robustness toward outliers. For multivariate analysis, a binary logistic regression analysis was performed due to an unfulfilled assumption of proportional odds in an ordinal logistic regression analysis. The effects of premature birth, UTI as first symptom, prenatal abnormal findings and gender (female gender as reference category) on the occurrence of CKD stage ≥ II were investigated. *P*-values of <0.05 were considered as significant.

## Results

### Study Population and Findings Related to CAKUT

**Figure 1:**
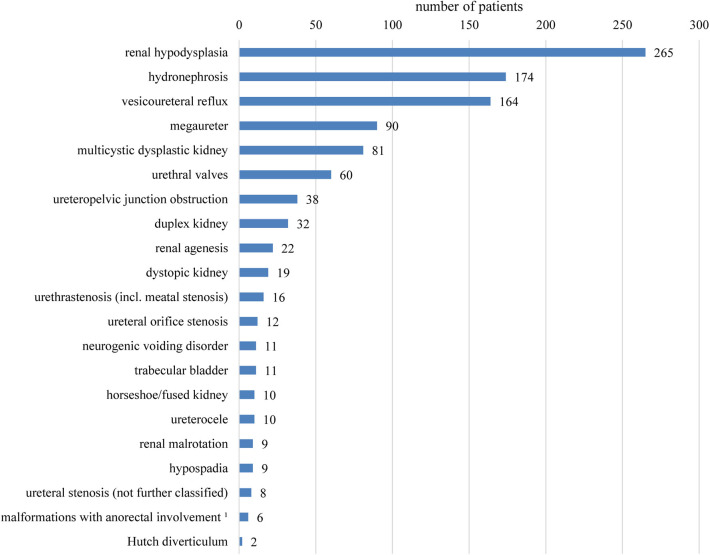
Overview of the registered findings affecting the kidneys and urinary tract. Several items per patient are possible. No differentiation between uni- or bilateral occurrence was made. 1Combined malformations of urinary tract and anus/rectum usually with fistula.

Table 1 summarizes basic data of the study population. 265 children (65.4%) had uni- or bilateral renal dysplasia/hypoplasia. Hydronephrosis was present in 174 patients (43.0%), VUR in 164 patients (40.5%), and megaureter in 90 patients (22.2%) (Figure 1). Identified syndromes associated with CAKUT are listed in Table 2. In the case of 38 of the 70 patients (54.3%) with syndromic diseases, the cluster of abnormalities could not be classified in a known syndrome up to now.

**Table 1:** Basic data of the study population.

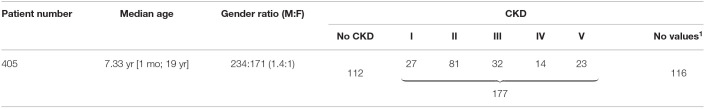

1 *Laboratory values for assessment of renal function not available*.

**Table 2:** Syndromes identified among the investigated patients with number of affected patients.

**Syndrome**	**Number of patients**
Renal cysts and diabetes syndrome	6
Branchiootorenal syndrome	3
Caudal regression syndrome	3
GATA3 mutation syndrome	3
Prune belly syndrome	3
Trisomy 21	2
Trichorhinophalangeal syndrome type I	2
Trisomy 18	1
Mayer-Rokitansky-Küster-Hauser syndrome	1
Kabuki syndrome	1
Wolf-Hirschhorn syndrome	1
Noonan syndrome	1
Williams-Beuren syndrome	1
Mabry syndrome	1
Myhre syndrome	1
VACTERL association	1
Deletion 22q13 syndrome	1
1q21.1 microdeletion syndrome	1

### First Symptoms

Overall, 189 patients (46.7%) were classified as prenatally abnormal because of prenatally seen abnormalities of the kidney or urinary tract (*n* = 187, 46.2%) and/or an oligo-/anhydramnios (*n* = 28, 6.9%). In 55 patients (13.6%), CAKUT was diagnosed in an ultrasound scan or voiding cystourethrography performed due to an UTI. Fifty-two patients (12.8%) were premature infants. None or other first symptoms (like another organ malformation) were mentioned in 150 patients (37%).

### Current Renal Function

**Figure 2:**
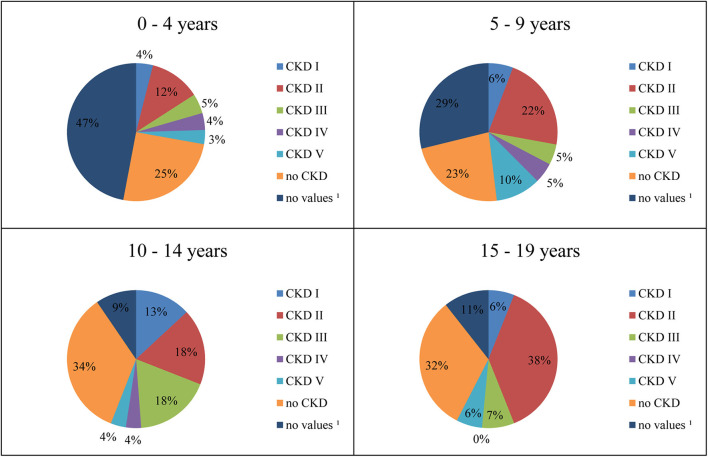
Distribution of CKD stages by age. 1Laboratory values for assessment of renal function not available.

One hundred seventy-seven patients (43.7%) were suffering from CKD (Table 1). In Figure 2, CKD stages in different age groups are shown.

### Factors Associated With Developing CKD

CKD stages were significantly higher in boys compared to girls (median CKD stage II vs. I, *p* = 0.004). This gender difference could also be observed when patients with syndrome associated CAKUT (*p* = 0.025) and patients with isolated CAKUT (*p* = 0.002) were analyzed separately.

Of the 189 patients with prenatal abnormal findings of the kidney or urinary tract or with an oligo-/anhydramnios, 86 patients (45.5%) showed CKD. In the group without documented prenatal findings, 91 (42.1%) had CKD. There was no statistically significant relationship between all CKD stages and presence of prenatal findings. However, children with prenatal abnormal findings showed more frequently a glomerular filtration rate (GFR) of <30 ml/min/1.73 m^2^ (18.9% vs. 7.6%, *p* = 0.004) compared to children without prenatal abnormalities. The children with prenatal findings were significantly younger (*p* < 0.001, median age 6 years and 11 months vs. 11 years and 2 months). In patients with isolated CAKUT, separately analyzed, the significant relationship between prenatal abnormal findings and a glomerular filtration rate (GFR) of <30 ml/min/1.73 m^2^ was also ascertained (*p* = 0.003). However, in patients with syndrome associated CAKUT a relationship between prenatal abnormal findings and CKD stages could not be observed.

Of the 55 patients diagnosed in the context of UTI, 14 patients (25.5%) had CKD. In contrast, 163 (46.6%) of the patients who were diagnosed independently of UTI had CKD. Patients with UTI as first symptom had significantly lower CKD stages than the other ones (median CKD stage 0 vs. II, *p* = 0.006). In patients with isolated CAKUT, CKD stages were significantly lower when UTI had occurred as first symptom (*p* = 0.01), too. In patients with syndrome associated CAKUT, no significant relationship could be observed.

Fifty-two patients (12.8%) were born premature. Of them, 36 (69.2%) had CKD. In the group of patients without reported prematurity, CKD was less common (141 patients out of 353, 39.9%). Children with premature birth had significantly higher CKD stages (median CKD stage II vs. I, *p* < 0.001). They were statistically significantly younger than the other ones at the reference date (median age 6 years and 10 months vs. 9 years and 9 months, *p* = 0.032). Among them, there were more males than females (74.5 vs. 56.6%, *p* = 0.022). In patients with isolated CAKUT, significant relationship between prematurity and CKD stages (*p* = 0.011) could be observed as well, but not in patients with syndrome associated CAKUT.

A binary logistic regression analysis on the occurrence of CKD stage ≥ II revealed that the model as a whole (*p* = 0.006) and the coefficients of the variables premature birth (*p* = 0.033) and UTI as the first symptom (*p* = 0.043) were significant (Table 3). The probability of occurrence of CKD stage ≥ II increased 2.2-fold in case of preterm birth. If UTI was the first symptom, the probability of occurrence of CKD stage ≥ II was 2.2 times lower. The variables prenatal abnormal findings and gender were not significant (Table 3).

**Table 3:** Binary logistic regression analysis of factors associated with CKD.

**Variable**	**OR**	**95% CI**	***p*-value**
Premature birth	2.153	1.065–4.354	0.033
UTI as first symptom	0.448	0.206–0.975	0.043
Gender (ref: female)	1.285	0.792–2.085	0.311
Prenatal abnormal findings	1.060	0.630–1.781	0.827

*OR, odds ratio; CI, confidence interval*.

### Secondary Findings and Diagnoses

The most frequently registered secondary finding was a patent foramen ovale (*n* = 28, 6.9%), followed by retained testis (*n* = 22, 9.4% of the boys) and patent ductus arteriosus (PDA, *n* = 20, 4.9%). In total, congenital structural cardiovascular anomalies were found in 57 patients (14.1%).

### Genetic Investigations

At least one genetic test was performed in 66 patients (16.3%). Twelve patients had pathological cytogenetic findings presumably or potentially being the genetic cause for CAKUT (Table 4). Molecular genetic findings that are presumably or potentially supposed to be a genetic cause for CAKUT were diagnosed in 18 patients (Table 4). Of these, more than one genetic aberration was detected in three patients.

**Table 4:** Genetic findings, separated in cytogenetic and molecular genetic.

**Cytogenetic findings**	**Number of patients**	**Molecular genetic findings**	**Number of patients**
**(A) Very likely or probably causative for renal phenotype**			
Trisomy 21	2	*HNF1$\underset{︸}{}\beta$*	6
Trisomy 18	1	*GATA3*	2
Unbalanced translocation chr 10; 16	1	*EYA1*	1
Microdeletion chr 8, suspected BOR syndrome	1	*KMT2D*	1
Microdeletion 22q13	1	*PAX2*	1
Microdeletion 1q21.1	1	*SMAD4*	1
Deletion 4p16.1, Wolf-Hirschhorn syndrome	1	*TRPS1*	2^1^
Deletion chr 7, Williams-Beuren syndrome	1	*PIGV*	1
**(B) Uncertain clinical relevance**			
Duplication chr 22	1	*FREM1*	1
3 duplications chr 1; 2	1	*GATA3*	1
Microdeletion chr 15	1	*FRAS1*	1
		*TFAP2A*	1
**(C) Probably without clinical relevance**			
Duplication chr 5	1	*RET*	2
		*ITGA8*	1
		*PAX2*	1
		*TBX18*	1
		*WWTR1*	1

1 *It is a pair of siblings, molecular genetic detection was performed in one of them and in the mother, in the second sibling diagnosis was made clinically because of these findings*.

Sequencing of genes being associated with CAKUT did not reveal any aberrations in 10 patients. One patient with an ascertained mutation in the gene *PKHD1* was suffering from an autosomal recessive polycystic kidney disease (ARPKD) and additionally from a double kidney with an ectopic entering ureter and a contralateral VUR. Since the appearance of CAKUT is not known in the context of ARPKD it is unlikely that the mutation causes the entire anomalies.

In another six cases, genetic testing was performed to rule out ciliopathies. The abnormalities of the patients did not seem to originate solely from detected aberrations in these tests, but a modifying effect cannot be excluded.

## Discussion

More than half of the children with ESRD suffer from a rare disease as underlying condition (5). Likewise, the importance of further research on rare kidney diseases is obvious. CAKUT are the heterogeneous rare disease group with the highest prevalence in children on renal replacement therapy (5).

In this study, 405 patients with CAKUT were investigated in detail regarding their pheno- and genotype. The most frequent finding related to CAKUT in the present study was renal dysplasia/hypoplasia, followed by hydronephrosis. In the literature, the rate of renal dysplasia/hypoplasia was lower, presumably because the diagnoses in these studies all were made prenatally or shortly after birth (15–17). Hydronephrosis was often the most common pathological finding in other studies too (13, 15–17). The high percentage of PUV (being diagnosed in 25.6% of the boys) explains the sex ratio of 57.8% boys to 42.2% girls of the entire study group. In previous publications, a similar ratio was reported (17–19).

43.7% of the children were suffering from CKD. The percentage of patients in CKD stages II and III is at the same level as in the studies of Soliman et al. (1) and Nef et al. (13). In the CKD stages IV and V, 9.1% of the patients were registered compared to 22.8% in the study of Soliman et al. (1) and 2.6% in the study of Nef et al. (13). A reason for the increased number of patients in high CKD stages reported by Soliman et al. may be a higher rate of PUV in their study (1, 13). Likewise, from other studies a high risk for development of CKD in boys with PUV is known (20–22). Male gender was associated with higher CKD stages in the univariate analysis. However, in the multivariate analysis, male gender in relation to the occurrence of CKD stage ≥ II did not yield a significant result. These differing results can probably be explained with regard to the detailed gender-specific distribution of the CKD stages: The boys had a relatively much higher proportion especially in the high CKD stages, but these are not high numbers in absolute terms.

The prevalence of premature births in Germany in 2008 was 9% of all live births (23). In the present study, 12.8% of the children were born prematurely. Therefore, a higher risk for premature birth in patients with CAKUT has to be assumed. Tain et al. identified a gestational age <37 weeks as a risk factor for occurrence of CAKUT (24). In general for children with congenital anomalies, a higher risk for prematurity is known, but the underlying cause is not fully understood (25). A likely explanation in the case of CAKUT is that an oligo-/anhydramnios is a known cause for early delivery.

Furthermore, the present study indicates premature birth in patients with CAKUT as a factor associated with developing CKD. Regarding potential factors influencing this outcome it has to be taken into account that there are two most likely contrary aspects with a higher rate of boys and a lower median age in the group of children with premature birth. Likewise, Nef et al. reported of a more frequent non-favorable outcome in patients with premature birth but their result was not statistically significant, maybe due to a low incidence of premature births (13). Oliveira et al. identified prematurity as an independent predictor of adverse outcome in children with fetal hydronephrosis (26). Interestingly, in patients with syndrome associated CAKUT premature birth was not a statistically significant factor associated with developing CKD.

In our study, 46.7% of the patients had prenatally abnormal ultrasound findings of the kidney or urinary tract and/or an oligo-/anhydramnios. This is in line with previously published studies [in 36.6% (1) and 56% of the patients (22)]. In the present study, a significant relationship between the presence of prenatal findings and a GFR of <30 ml/min/1.73 m^2^ was identified. This result is supported by the study of Nef et al., where they identified oligohydramnios as a factor associated with poor outcome (13). A pathological prenatal ultrasound may also reflect the severity of the malformation. Furthermore, it has to be taken into account that in the present study some patients with prenatal findings might have been ranged in the group without those findings by mistake, due to missing data. Another aspect to be considered for the interpretation of the result is the significantly lower age in the group of children with prenatal findings, particularly as the prevalence of CAKUT patients with CKD rises with increasing age. Thus, we suspect an even stronger relationship if patients of both groups were the same age.

UTIs were the reason for ultrasound examination or voiding cystourethrography that revealed CAKUT in more than 10% of the patients. The significant negative relationship between CKD stages and UTIs as a cause of initial presentation, especially in children with isolated CAKUT, suggests that children diagnosed with having UTI have a better prognosis in comparison to the other patients. A reason for this might be the circumstance that the patients were clinically unremarkable until UTI.

Overall, the percentage of patients with cardiac anomalies was much higher than in the general population with for instance higher rates of ventricular septal defects and atrial septal defects (27). This is in line with the results of the study of Stoll et al. (17). A reason for a higher prevalence of congenital heart defects in patients with CAKUT is presumably the occurrence of CAKUT within syndromes. In practice, patients with extrarenal anomalies have to be identified. For their care it has to be taken into account that the presence of extrarenal abnormalities is associated with an impaired outcome, at least in the neonatal period (28, 29).

In this study, the disease pattern of 17.3% of the children were classified with syndromic disease. In the study of Stoll et al. in 34% an “associated CAKUT” was diagnosed (17). Stillbirths and terminations of pregnancy after prenatal diagnosis and thus children with fatal abnormalities were included in that study which may explain a higher rate of syndromes (17). Notably, the identified factors associated with developing CKD, except for gender, could only be observed in isolated CAKUT cases. In patients with syndrome associated CAKUT, a relationship between prematurity, prenatal pathological findings or UTI as first symptom could not be confirmed. This may be due to the small number of patients with syndrome associated CAKUT and the resulting low statistical power.

In 30 patients, genetic aberrations presumably or potentially supposed to be a genetic cause for CAKUT were found, in 12 patients as cytogenetic and in 18 patients as molecular genetic mutations. Altogether, the detection rate (45%) was quite high compared to previously published studies. Hwang et al. identified in 6.3% patients with isolated CAKUT a mutation in a known CAKUT associated gene (18). In the study of Lei et al., the detection rate in fetuses with normal karyotype and chromosome microarray analysis was 9.1% for isolated CAKUT and 25% for CAKUT in combination with other abnormalities (30). The reason for the higher detection rate in the present study might be due to preselection of the children for genetic testing who had in many cases severe CAKUT malformations and associated abnormalities.

The most common identified cytogenetic correlate to the phenotype was trisomy 21 with a similar rate as previously reported by Melo et al. (28). At molecular genetic level, mutations in the gene *HNF1*β were most frequently found in 1.5% of the patients. Mutations in the *HNF1*β or in the *PAX2* gene are reported as common causes of CAKUT. In the study of Hwang et al. the prevalence of mutations in *HNF1*β and *PAX2* genes are comparable to this study (18). In the studies of Weber et al. and Thomas et al., prevalence was even higher (31, 32). This could be due to the fact that only patients with CKD and renal hypodysplasia were included (31, 32).

Concerning the genetic results, one should be aware that the molecular genetic testing included only a sequencing of genes being associated with CAKUT or an assumed syndrome. The pathogenicity of gene aberrations is currently difficult to define without functional assays. Aberrations are therefore classified for instance as “pathogenic effect quite conceivable” or “undetermined pathogenic” by the human geneticists. Similarly, a connection between a diagnosed syndrome and existent CAKUT is not always directly done, if CAKUT do not belong typically to the underlying syndrome.

Considering limitations of the present study, there are mainly two: Firstly, it is a monocentric study in a tertiary center. Thus, it is not ensured that the results are applicable to the general population. Secondly, data was gathered retrospectively from patient charts as a cross-sectional study, explaining why data was often not available for all parameters and a possible later progress toward CKD in some patients has not yet occurred. Besides, a time-to-event analysis, which could provide relevant additional results, was not possible. There are prospective monocentric studies published, but most of them have a different focus or the investigated population differs presumably a lot, like in the study of Soliman et al. (1). Moreover, the number of patients being included in other published studies was distinctly lower (1, 13, 15, 16, 22). Furthermore, for the interpretation of the results of the present study it should be considered that in 28.6% of the patients no laboratory values were available for the assessment of renal function and therefore these patients were not included in the analysis of possible factors associated with CKD.

In conclusion, this study provides an informative characterization of a large patient group with CAKUT. Potential significant factors associated with a poor outcome, like gender, prematurity and prenatal abnormal findings, were identified. The results should be validated in prospective studies. Close follow-up visits in accordance to the identified factors are recommended as CAKUT represent the most common cause of CKD in children and adolescents.

## Data Availability Statement

The raw data supporting the conclusions of this article will be made available by the authors, without undue reservation.

## Ethics Statement

The studies involving human participants were reviewed and approved by Ethic Committe Charité. Written informed consent to participate in this study was provided by the participants' legal guardian/next of kin.

## Author Contributions

SI, DM, and JT: conceptualization and validation. SI: methodology, formal analysis, writing-original draft preparation, and visualization. DM and JT: writing-review and editing. DM: supervision.

## Conflict of Interest

The authors declare that the research was conducted in the absence of any commercial or financial relationships that could be construed as a potential conflict of interest.
